# Investigation of bedaquiline resistance and genetic mutations in multi-drug resistant *Mycobacterium tuberculosis* clinical isolates in Chongqing, China

**DOI:** 10.1186/s12941-023-00568-0

**Published:** 2023-02-28

**Authors:** Yan Hu, Jun Fan, Damin Zhu, Wenguo Liu, Feina Li, Tongxin Li, Huiwen Zheng

**Affiliations:** 1 Tuberculosis Reference Laboratory, Chongqing Tuberculosis Control Institute, Chongqing, China; 2 Laboratory of Respiratory Diseases, Beijing Key Laboratory of Pediatric Respiratory Infection Diseases, Beijing Pediatric Research Institute, Key Laboratory of Major Diseases in Children, Ministry of Education, National Clinical Research Center for Respiratory Diseases, Beijing Children’s Hospital, Capital Medical University, National Center for Children’s Health, Beijing, 100045 China; 3 Central Laboratory, Chongqing Public Health Medical Center, Chongqing, 400036 China

**Keywords:** Bedaquiline, Mutation, Resistance, MDR

## Abstract

**Background:**

To investigate the prevalence and molecular characterization of bedaquiline resistance among MDR-TB isolates collected from Chongqing, China.

**Methods:**

A total of 205 MDR-TB isolates were collected from Chongqing Tuberculosis Control Institute between March 2019 and June 2020. The MICs of BDQ were determined by microplate alamarblue assay. All strains were genotyped by melting curve spoligotyping, and were subjected to WGS.

**Results:**

Among the 205 MDR isolates, the resistance rate of BDQ was 4.4% (9/205). The 55 (26.8%) were from male patients and 50 (24.4%) were new cases. Furthermore, 81 (39.5%) of these patients exhibited lung cavitation, 13 (6.3%) patients afflicted with diabetes mellitus, and 170 (82.9%) isolates belonged to Beijing family. However, the distribution of BDQ resistant isolates showed no significant difference among these characteristics. Of the 86 OFX resistant isolates, 8 isolates were XDR (9.3%, 8/86). Six BDQ resistant isolates (66.7%, 6/9) and two BDQ susceptible isolates (1.0%, 2/196) carried mutations in *Rv0*678. A total of 4 mutations types were identified in BDQ resistant isolates, including mutation in A152G (50%, 3/6), T56C (16.7%, 1/6), GA492 insertion (16.7%, 1/6), and A274 insertion (16.7%, 1/6). BDQ showed excellent activity against MDR-TB in Chongqing.

**Conclusions:**

BDQ showed excellent activity against MDR-TB in Chongqing. The resistance rate of BDQ was not related to demographic and clinical characteristics. Mutations in *Rv*0678 gene were the major mechanism to BDQ resistance, with A152G as the most common mutation type. WGS has a good popularize value and application prospect in the rapid detection of BDQ resistance.

## Introduction

Drug-resistant tuberculosis, especially multidrug-resistant tuberculosis (MDR-TB), remains a major threat to global TB control and prevention strategy. In 2020, an estimate of approximate 0.5 million rifampicin-resistant (RR-)/MDR-TB cases occurred globally, of which 78% were MDR-TB [1]. The treatment of MDR-TB is challenging due to the lack of effective drugs, and the overall rate of treatment success is currently 57%, imposing a burden on health care resources [1]. Therefore, new and effective anti-TB drugs are urgently needed to improve the chemotherapy of MDR-TB [2].

Bedaquiline (BDQ), a novel oral diarylquinoline drug, had excellent efficacy against both drug susceptible and drug resistant MTB [3] and was recommended by WHO for the treatment of MDR [4]. However, BDQ resistance was also emerged with the introduction to the treatment regimens, and several mechanisms of BDQ resistance had been identified. Mutations in *atpE* gene, encoding subunit C of the ATP synthase, can prevent BDQ from binding to the C subunit, thus resulting in BDQ resistance [3]. Mutations in *Rv*0678 gene, coding for the repressor of MmpS5-MmpL5 efflux system, were associated with resistance to BDQ [5, 6]. Besides, mutations in gene encoding the uncharacterized transporter Rv1979c and the cytoplasmic peptidase PepQ were also reported to confer BDQ resistance [7–9].

Though BDQ has not been widely used in China, the primary drug resistance of BDQ has emerged [6]. Chongqing, the only municipal city in Southwest China with a high incidence of tuberculosis, will promote the use of BDQ in the treatment of MDR-TB. However, with little information about the prevalence of BDQ resistance in Chongqing, it is meaningful to investigate the prevalence and molecular characterization of BDQ resistance by whole genome sequencing (WGS) among MDR-TB isolates, which will improve the diagnosis and treatment of MDR patients.

## Materials and methods

### Bacterial strains

A total of 205 MDR-TB isolates were collected from Chongqing Tuberculosis Control Institute between March 2019 and June 2020. All isolates were from patients with symptoms suggestive of active pulmonary TB, and the demographic and clinical characteristics were obtained. All isolates were subcultured on the Löwenstein–Jensen (L–J) medium for 4 weeks at 37 ℃.

### Conventional drug susceptibility testing

Drug susceptibility was determined using the 1% proportion method on L–J medium according to the guidelines of the WHO [10], with rifampin (RIF), 40 μg/ml; isoniazid (INH), 0.2 μg/ml; streptomycin (SM), 10 μg/ml; ethambutol (EMB), 2 μg/ml; capreomycin (CM), 40 μg/ml; kanamycin (KM), 30 μg/ml; ofloxacin (OFX), 2 μg/ml; amikacin (AM). The MDR-TB was defined as resistance to at least INH and RIF. Extensively drug-resistant tuberculosis (XDR-TB) isolates were defined as MDR-TB isolates with additional resistance to both OFX and KM [11].

### Minimum inhibitory concentrations

For MDR-TB identified by conventional drug susceptibility testing, the MICs of BDQ were determined using microplate alamarblue assay [12]. The breakpoint concentrations were defined as 0.25 μg/ml for BDQ according to the European Committee on Antimicrobial Susceptibility Testing (EUCAST) guidelines [13, 14]. Mycobacterium tuberculosis H37Rv (ATCC 27249) was used as the control strain. The MIC value was defined as the lowest concentration of antibiotic that inhibited visible growth of mycobacteria. MIC_50_ and MIC_90_ was defined as the concentration required to inhibit the growth of 50% and 90% of the strains, respectively.

### MeltPro assay

Genomic DNA from MDR-TB isolates was extracted using the cetyltrimethylammonium bromide (CTAB) method. All strains were genotyped by melting curve spoligotyping performed in the SLAN-96S system (Hongshi, Shanghai, China) as previously described [15]. The results were automatically exported by the SLAN software (Zeesan, Xiamen, China), followed by comparing to the SITVIT database to identify the genotype.

### Whole genome sequencing

The qualified DNA samples were sent to the Annoroad Gene Technology (Beijing, China) for whole genome sequencing (WGS) service based on Illumina Hiseq2500 sequencing platform. The sequencing reads were aligned to the H37Rv reference genome (NC_000962).

### Statistical analysis

The person chi-square test or Fisher exact test was used to compare proportions or resistant rates. A *P* < 0.05 was considered statistically significant. All the statistical analyses were performed in the SPSS 20.0 (IBM Corp., Armonk, NY).

## Results

### BDQ MIC to MDR

**Fig. 1:**
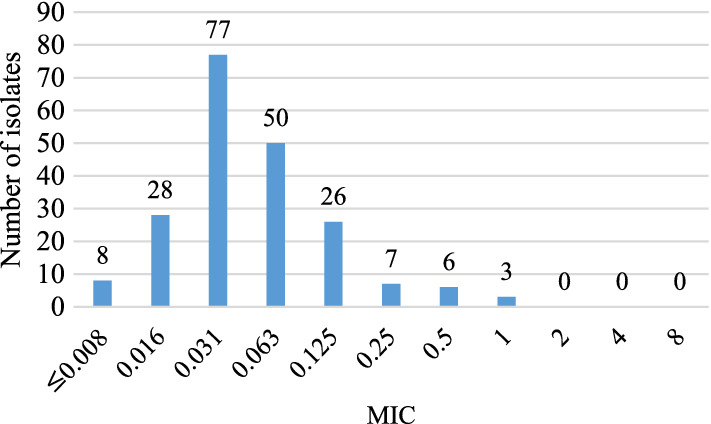
Distribution of minimum inhibitory concentration (MIC, μg/ml) of BDQ for MDR (n = 205)

The distribution of MDR isolates at the MIC of BDQ was shown in Fig. 1. Among the 205 MDR isolates, the number of bacteria showing MIC > 0.25 μg/ml as determined by BDQ resistance was 4.4% (9/205). The MIC_50_ and MIC_90_ values were 0.031 μg/ml and 0.125 μg/ml, respectively.

### Clinical data analysis of MDR isolates

Demographic and clinical characteristics of MDR isolates patients were summarized in Table 1. For the 205 MDR patients, 55 (26.8%) were from female patients, and there were 50 (24.4%) new cases and 155 (75.6%) re-treated cases. The resistance rate of BDQ (4.4%, 9/205) was lower than that of commonly used first- and second-line drugs with SM (72.2%, 148/205), EMB (37.6%, 77/205), OFX (42.0%, 86/205) and KM (14.6%, 30/205). Of the 9 BDQ resistant isolates, the proportion of ancient Beijing strains (88.9%, 8/9) was significantly higher than that of modern Beijing strains (11.1%, 1/9) (*P* < 0.01), and the number of OFX resistant isolates was significantly higher than that of OFX sensitive isolates (*P* < 0.01).

**Table 1:** Differences of characteristics between BDQ^R^ and BDQ^S^ MDR strains

Characteristics	No. (%) of isolates (n = 205)	No. (%) of isolates			
		BDQ^R^ (*n* = 9)	BDQ^S^ (*n* = 196)	OR	*P*
				(95%CI)	
Sex					
Female	55(26.8)	3(33.3)	52(26.5)	Ref.	
Male	150(73.2)	6(66.7)	144(73.5)	0.72(0.17–2.99)	0.95
Age (years)					
≤ 40	69(33.7)	4(44.4)	65(33.2)	Ref.	
41–59	96(46.8)	3(33.3)	93(47.4)	0.52(0.11–2.42)	0.65
≥ 60	40(19.5)	2(22.2)	38(19.4)	0.86(0.15–4.89)	1.00
Lineage					
Lineage 4	37(18.0)	0(0.0)	37(18.9)	Ref.	
Lineage 2	168(82.0)	9(100.0)	159(8.1)	0.95(0.91–0.98)	0.32
Genotype					
Modern Beijing	98(47.8)	1(11.1)	97(49.5)	Ref.	
Ancient Beijing	72(35.1)	8(88.9)	64(32.7)	12.13(1.48–99.28)	< 0.01
Non-Beijing	35(17.1)	0(0.0)	35(17.8)	1.01(0.99–1.03)	1.00
Treatment History					
New case	50(24.4)	3(33.3)	47(24.0)	Ref.	
Re-treated	155(75.6)	6(66.7)	149(76.0)	0.63(0.15–2.62)	0.81
Lung Cavitation					
No	124(60.5)	6(66.7)	118(60.2)	Ref	
Yes	81(39.5)	3(33.3)	78(39.8)	0.76(0.18–3.11)	0.97
Diabetes Mellitus					
No	192(93.7)	7(77.8)	185(94.4)	Ref.	
Yes	13(6.3)	2(22.2)	11(5.6)	4.81(0.89–25.90)	0.10
Previous exposure to					
None	16(7.8)	1(11.1)	15(7.7)	Ref.	
FL drugs	124(60.5)	5(55.6)	119(60.7)	0.63(0.07–5.76)	0.52
FL and SL drugs	65(31.7)	3(33.3)	62(31.6)	0.73(0.07–7.48)	1.00
Resistance to					
SM	148(72.2)	7(77.8)	141(71.9)	4.81(0.89–25.91)	0.10
EMB	77(37.6)	5(55.6)	72(36.7)	2.15(0.56–8.28)	0.43
OFX	86(42.0)	8(88.9)	78(39.8)	12.10(1.48–98.68)	< 0.01
KM	30(14.6)	1(11.1)	29(14.8)	0.72(0.09–5.97)	1.00

### MDR against BDQ in different resistance pattern

The MIC of BDQ resistant isolates against SM, EMB, KM and OFX was shown in Table 2. The 31 isolates sensitive to SM, EMB, KM and OFX were all susceptibility to BDQ. As the number of drug resistance increases, the drug resistance rate of BDQ increased from 0 to 14.4%. Of the 86 OFX resistant isolates, 8 isolates were XDR with the resistance rate of 9.3% (8/86). And the resistance rate of BDQ in OFX resistant isolates (9.3%) was higher than that in SM resistant isolates (4.7%), EMB resistant isolates (6.5%), and KM resistant isolates (3.3%). The resistance rate of BDQ in isolates resistant to any first and second line drug (8.9%) was higher than that in isolates resistant to first line drugs (7.7%) and second line drugs (1.2%), respectively.

**Table 2:** MIC distribution of BDQ resistant isolates against SM, EMB, KM and OFX

Drug resistance profile	No. of strains	No. of strains with different MIC (μg/ml)											No. (%) of BDQ resistant strains
		≤ 0.008	0.016	0.031	0.063	0.125	0.25	0.5	1	2	4	8	
All isolates	205	8	28	77	50	26	7	6	3	0	0	0	9 (4.4)
Fully susceptible isolates	31	2	6	13	7	1	2	0	0	0	0	0	0(0)
Resistant to one drug	56	4	5	22	15	7	2	1	0	0	0	0	1(1.8)
Resistant to two drugs	76	1	11	30	17	11	3	3	0	0	0	0	3 (3.9)
Resistant to three drugs	35	1	4	11	9	5	1	2	2	0	0	0	4(11.4)
Resistant to four drugs	7	0	2	1	2	1	0	1	0	0	0	0	1(14.4)
Resistant to SM	148	3	18	57	35	23	5	6	1	0	0	0	7(4.7)
Resistant to EMB	77	2	9	28	17	12	3	4	1	0	0	0	5(6.5)
Resistant to OFX	86	2	13	27	23	11	2	6	2	0	0	0	8(9.3)
Resistant to KM	30	2	7	7	9	2	1	1	0	0	0	0	1(3.3)
Resistant to SM and (or) EMB	82	4	8	35	18	13	3	1	0	0	0	0	1(1.2)
Resistant to KM and (or) OFX	13	1	3	3	4	1	0	1	0	0	0	0	1(7.7)
Resistant to any first and second line drugs	79	1	11	26	21	10	3	5	2	0	0	0	7(8.9)

### WGS Identification of BDQ resistance-related mutations

The BDQ-resistant mutants were performed by WGS in 205 MDR isolates (Table 3). No mutations within the *atpE*, *pepQ*, and *Rv1979* gene were observed in 9 BDQ resistant isolates. Six BDQ resistant isolates (66.7%, 6/9) and two BDQ susceptible isolates (1.0%, 2/196) carried mutations in *Rv*0678, which has statistical significance. A total of 4 mutations types were identified in BDQ resistant isolates, including A152G mutation causing Gln51Arg amino acid change (50%, 3/6), T56C mutation causing Phe19Ser amino acid change (16.7%, 1/6), GA492 insertion (16.7%, 1/6), and A274 insertion (16.7%, 1/6). Besides, G307A causing Gly103Ser amino acid change and G184A causing Ala62Thr amino acid change in the *Rv0678* gene were identified in BDQ sensitive isolates. The six BDQ resistant isolates with mutations in *Rv0678* gene all belonged to ancient Beijing genotype, and were resistant to at least two drugs in Table 4. Both of the two BDQ susceptible isolates with mutations in *Rv0678* gene, one non-Beijing and one mordern Beijing genotype, were resistant to SM.

**Table 3:** Mutation analysis of BDQ resistant genes among 205 MDR isolates

Resistance pattern	Isolate number	Gene mutation type				No. of isolates (%)	MIC of BDQ (μg/ml)
		*atpE*	*Rv0678*	*pepQ*	*Rv1979*		
BDQ resistant isolates (9)	22A050, 22A133, 22A148	WT	CAG152CGG Gln51Arg	WT	WT	3	0.500
	22A118	WT	TTC56TCC Phe19Ser	WT	WT	1	0.500
	22A177	WT	492 position ins_GA	WT	WT	1	1.000
	22A180	WT	274 position ins -A	WT	WT	1	1.000
Total						6 (66.7)^a^	0.500–1.000
BDQ sensitive isolates (196)	22A076	WT	GGC307AGC Gly103Ser	WT	WT	1	0.125
	22A079	WT	GCC184ACC Ala62Thr	WT	WT	1	0.250
	22A128	WT	WT	GCC411GCT Ala137Ala	WT	1	0.031
	22A174	WT	WT	GAA1080GAT/Glu360Asp	WT	1	0.016
	22A012, 22A025, 22A030, 22A032, 22A041	WT	WT	WT	GTT1276ATT/Val426lle	5	0.031–0.063
	22A222	WT	WT	WT	C(−70)G	1	0.063
	22A196	WT	WT	WT	GCG717GCA/Ala239Ala	1	0.063
	22A005	WT	WT	WT	TCG785TTG/Ser262Leu	1	0.016
	22A227	WT	WT	WT	GTC286ATC/Val96lle	1	0.031
	22A204	WT	WT	WT	GCC449GTC/Ala150Val	1	0.250
	22A039	WT	WT	WT	GTT155GGT/Val52Gly	1	0.031
	22A016, 22A029, 22A201, 22A220, 22A223	WT	WT	WT	WT	5	≤ 0.008–0.031
	22A006, 22A009	WT	WT	WT	WT	2	0.031
	22A044	WT	WT	WT	WT	1	0.016
	22A206	WT	WT	WT	WT	1	0.031
	22A028	WT	WT	WT	WT	1	≤ 0.008
	22A008	WT	WT	WT	WT	1	0.031
Total						26 (13.3)^b^	≤ 0.008–0.250

Compared a to b: *X*^2^ = 14.795

*P* < 0.001

**Table 4:** Drug resistance data of isolates with mutations in *Rv0678* gene

Isolate number	MIC of BDQ (μg/ml)	Drug resistance profile	Genotype
22A050	0.5	SM + EMB + OFX	Ancient Beijing
22A118	0.5	SM + OFX	Ancient Beijing
22A133	0.5	SM + EMB + OFX	Ancient Beijing
22A148	0.5	SM + EMB + OFX + KM	Ancient Beijing
22A177	1	EMB + OFX + KM	Ancient Beijing
22A180	1	SM + EMB + OFX	Ancient Beijing
22A076	0.125	SM	non-Beijing
22A079	0.25	SM	Mordern Beijing

### Genotypic predictions

As shown in Table 5, the sensitivity of WGS prediction for BDQ resistance was 66.7%, the specificity was 99.0%, the positive predictive value was 75.0%, and the negative predictive value was 98.5%.

**Table 5:** WGS predictions versus DST phenotype for BDQ

WGS	DST phenotype (n)		Total	Sensitivity (%)	Specificity (%)	PPV (%)	NPV (%)	Kappa
	Resistant(9)	Sensitive(196)						
Mutation	6	2	8	66.7(30.9–91.0)	99.0(96.0–99.8)	75.0(35.6–95.5)	98.5(95.3–99.6)	0.693
Non-mutaion	3	194	197					

## Discussion

Although BDQ has been proven to be highly effective in the treatment of MDR-TB [16], inadequate or incomplete use may lead to the emergence of resistant strains [17]. Unfortunately, few studies have explored the resistance status of MDR-TB against BDQ in Chongqing. Therefore, we performed drug susceptibility test and conducted sequence analyses of BDQ resistance genes for 205 MDR isolates. The resistance rate of MDR-TB to BDQ was 4.4%, lower than that of commonly used first- and second-line drugs, indicating that BDQ has strong activity against MDR isolates in Chongqing. Though the resistance rate lower than that reported in Shanxi (5.56%) [15] and in national survey in China (7.16%) [18], higher than reported in a retrospective cohort study in China (2.2%) [19] and national drug resistance surveillance in 2015 (1%) [20]. These inconsistent results may be attributed to the difference in the epidemic strains, medication background and the breakpoints used across studies. Given the cross resistance between BDQ and clofazimine, prior exposure to clofazimine could reduce the susceptibility to BDQ [21]. And the period from the start of treatment can also affect the BDQ MIC [22]. To our knowledge, all isolates were without documented prior use of BDQ, and 4.4% MDR-TB strains resistant to BDQ suggesting that though BDQ showed excellent activity against MDR-TB, the emergence of BDQ resistant isolates may lead to the rapid loss of this valuable new drug. Therefore, it is necessary to dynamically monitor the BDQ resistance to optimize BDQ administration regimen, further to avoid the occurrence of acquired resistance, and maximize the effectiveness of new drugs, even in patients who have not been exposed to BDQ.

The resistance rate of BDQ in isolates resistant to any first and second line drug (8.9%) was higher than that in isolates resistant to first line drugs (1.2%) and second line drugs (7.7%), indicating that with the increase of drug resistance types and the complexity of resistant background, the BDQ resistance rate also increased. In addition, we found that the BDQ resistance rate in retreated patients (66.7%) was higher than that of new patients (33.3%), whether this attributed to the past medical history needs to be further studied. Of the 9 BDQ resistant isolates, the proportion of OFX resistant isolates (8/9) was significantly higher than that of OFX sensitive isolate (1/9), and the resistance rate of BDQ in OFX resistant isolates (9.3%) was higher than that in SM resistant isolates (4.7%), EMB resistant isolates (6.5%), KM resistant isolates (3.3%), suggesting isolates resistant to OFX were more likely to develop BDQ resistance, which was a risk factor of BDQ resistance.

Since the development and approval of BDQ for clinical use, the number of BDQ resistant isolates associated with inadequate or incomplete treatment is steadily growing [22]. To investigate the potential mechanisms and genetic background of BDQ resistant isolates, we performed whole-genome sequencing. Though the fact that mutations in the *atpE*, *pepQ*, and *Rv1979c* gene confer bedaquiline resistance [3, 7, 8], no mutations were observed in this study. The 66.7% (6/9) BDQ resistant isolates had variants in the *Rv0678* gene, which was the main mechanism of primary BDQ resistance in Chongqing, and all belonged to low level resistance (0.5–1 μg/ml). The mutation loci in *Rv*0678 gene were scattered and the mutation types were complicated. Of the 6 isolates carrying *Rv*0678 mutations included two non-synonymous Single Nucleotide Polymorphisms SNPs and deletions, the most frequently variations were A152G (50%), which has reported to be associated with BDQ resistance in MDR isolates [23]. Besides, the A274 insertion identified in the present study was found in clinical BDQ-resistant isolates [6]*.* However, there were three BDQ resistance isolates (33.3%, 3/9) without mutations, suggesting additional mechanisms must be involved in the resistance, such as other potential target and non-target resistance mechanisms, such as changes in cell wall permeability caused by transcriptional and protein levels and drug efflux pump structure. Two BDQ susceptible isolates with mutations in *Rv0678* gene were in the critical concentration of BDQ resistance and a gradient below the critical concentration, which may be attributed to operational factors, such as result interpretation, bacteria activity, drug concentration or other inaccurate factors. Two *pepQ* mutant strains and 11 *Rv1979* mutant strains were all sensitive to BDQ, which were not related to drug resistance. Moreover, the other two (*Rv0678* T56C and GA492 insertion) were novel mutation types, which were not reported previously. Further analysis in expression levels of MmpS5 and MmpL5 efflux pump will contribute to illustrate the role of these novel mutations in BDQ resistance.

The Beijing genotype was the predominant isolates in Chongqing with 47.8% modern Beijing genotype and 35.1% ancient Beijing genotype. However, the proportion of ancient Beijing strains (88.9%, 8/9) was significantly higher than that of modern Beijing strains (11.1%, 1/9) in BDQ resistant isolates, and 75% (6/8) BDQ resistant isolates with *Rv*0678 mutation were ancient Beijing type, indicating ancient Beijing genotype was more prone to BDQ resistance and *Rv*0678 mutation.

In this study, WGS for BDQ drug resistance was consistent with phenotypic drug susceptibility test. However, the relatively dispersed mutation loci of BDQ resistance associated genes may result in the presence of "false-susceptible" detected by PCR-sequencing of hot spots of current resistance-associated genes. Therefore, WGS can quickly and accurately determine the mutation loci, and has preferable specificity (99%) in predicting BDQ resistance. But for the non-target resistance mechanism, the phenotypic drug sensitive test was superior to WGS. So, the phenotypic drug sensitive test together with WGS was helpful to early diagnosis and individualized treatment of drug-resistant tuberculosis, which has excellent application value in the rapid detection of BDQ resistance.

## Conclusions

BDQ showed excellent activity against MDR-TB in Chongqing. The resistance rate of BDQ was not related to demographic and clinical characteristics. Mutations in *Rv*0678 gene were the major mechanism to BDQ resistance, with A152G as the most common mutation type. WGS has a good popularize value and application prospect in the rapid detection of Bdq resistance.

## Data Availability

The datasets in the present study are accessible from the corresponding author, ZHENG HW.

## Abbreviations

MDR-TB: Multidrug-resistant tuberculosis
RR: Rifampicin resistant
BDQ: Bedaquiline
WGS: Whole genome sequencing
L–J: Lowen Löwenstein–Jensen
RIF: Rifampin
INH: Isoniazid
SM: Streptomycin
EMB: Ethambutol
CM: Capreomycin
KM: Kanamycin
OFX: Ofloxacin
AMK: Amikacin
XDR-TB: Extensively drug-resistant tuberculosis
EUCAST: European Committee on Antimicrobial Susceptibility Testing
CTAB: Cetyltrimethylammonium bromide
